# Evacuation from Healthcare Facilities in Poland: Legal Preparedness and Preparation

**DOI:** 10.3390/ijerph17051779

**Published:** 2020-03-09

**Authors:** Krzysztof Goniewicz, Patrycja Misztal-Okońska, Witold Pawłowski, Frederick M. Burkle, Robert Czerski, Attila J. Hertelendy, Mariusz Goniewicz

**Affiliations:** 1 Department of Aviation Security, Polish Air Force Academy, 08-521 Dęblin, Poland; r.czerski@law.mil.pl; 2 Department of Emergency Medicine, Medical University of Lublin, 20-059, Lublin, Poland; patrycja.okonska@umlub.pl (P.M.-O.); witold.pawlowskl@dr.com (W.P.); mariusz.goniewicz@umlub.pl (M.G.); 3 Harvard Humanitarian Initiative, T.H. Chan School of Public Health, Harvard University, Boston, MA 02115, USA; skipmd77@aol.com; 4 Department of Information Systems and Business Analytics, College of Business, Florida International University, Miami, FL 33174, USA; attila.hertelendy@georgetown.edu

**Keywords:** evacuation, healthcare facilities, hospital safety, legal preparedness

## Abstract

Medical facilities, while providing both essential and demanding health care to society’s most vulnerable populations, also belong to the most demanding category of risk to human life if and when a crisis event occurs within its walls. The development of a safe evacuation plan for these facilities is extremely complicated, as the evacuation of medical facilities is much more complex than for other critical infrastructure. In this category, the evacuated patients constitute a specific risk group requiring specialized medical care. Hospitalized persons may be dependent on life-saving measures, are unconscious or immobile, are significantly restricted in movement or mentally unbalanced, being dependent on the continued assistance of trained third parties. Additionally, the medical transport of evacuated patients becomes more difficult due to the limited capacity of ambulances and available health care facilities to transport them to, which are increasingly limited due to their overcrowded census. The study aimed to analyze the requirements which are placed on hospitals in Poland to ensure the safety of patients in case of an evacuation. The research method used in the paper was retrospective analysis and evaluation of the media and literature. We have found, that Polish law imposes an obligation on the administrator of a medical facility to ensure the safety of both patients and employees. The regulations cover issues of technical conditions to be met by buildings and their location, prevention, and fire protection requirements, and the determination of which staff is responsible for the evacuation. However, available documents fail to describe what the hospital evacuation process itself should entail under emergency evacuation. Taking into account the complexity of the hospital evacuation process, health care facilities should have a well-developed plan of action that must be implemented at least once a year in the form of facility-wide training. Evacuation drills should not be avoided. Only trained procedures offer the possibility of later analysis to identify and eliminate errors and provide the opportunity to acquire skill sets and habits which promote the behaviors expected in real-life emergencies.

## 1. Introduction

Following the regulations in force in Poland, there are five categories of human hazards (*human hazards – HH*), which are defined by codes from abbreviations HH I to HH V [1]. Hospitals, according to technical conditions, belong to the most demanding category of risk to human life HH II, which includes all healthcare facilities intended primarily for use by people with reduced mobility (e.g. kindergartens, hospitals, nursing homes, retirement homes, hospices, etc.).

Every public building must have a procedure in case of an evacuation: whether it is a fire or a bomb alarm. Various scenarios are run. In the event of a fire, the entire wing or building will have to be evacuated, in the event of flooding, patients and equipment will need to be moved to higher floors. In the event of a biological emergency, hospital isolations are warranted. When creating evacuation plans, the area, infrastructure, and resources at the hospital’s disposal are taken into account. How many ambulances can be spent on transporting patients to t to other hospitals? How many people from the hospital can be delegated to lead able patients to a safe place? The personnel of the facility is also divided in advance into task teams with specific tasks assigned to them. The action is supervised by the hospital management in consultation with the firefighters, police and voivode (the regional representatives of the governmental administration).

Such facilities are subject to additional technical requirements that impact success or failure in case of a sudden evacuation. To highlight just some examples, legal restrictions require that the length of the evacuation access in one direction may not exceed 10 meters. In the case of existing facilities, a twofold increase in this length may constitute the basis for classifying the facility as life-threatening during any evacuation. Also, the same regulations require that the load-bearing capacity of the structure must be ensured for the entire time of evacuation, the facility must be protected against the spread of fire and smoke in all sections of the building (staircases must be separated from other spaces, closed by doors and smoke vented or protected against smoke), the spread of fire to adjacent buildings must be limited, people must be able to evacuate to safe accommodations, and the safety of rescue crews must be safeguarded. 

The evacuation process requires a significant amount of human resources and takes a long time. Complete hospital evacuations due to natural or man-made disasters can have repercussions on all levels of hospital operations. An extended displacement period following an evacuation exacerbates the situation [2].

## 2. Material and Methods

A retrospective analysis was conducted incorporating media and a literature review. This retrospective analysis was performed using local press resources and other media reports to find evacuation evens in every province of Poland since 2012. The collected data included the type of event which forced an evacuation action. False alarms were most often recorded and didn’t trigger evacuation, hence, they were not captured in our data set. Investigators also reviewed legal acts and articles. These articles and acts were selected if they contained information pertaining to a hospital response to an evacuation.

## 3. Evacuation of Hospital Facilities in Poland

A retrospective analysis of data on the causes of the evacuation of HH II facilities (hospitals and nursing homes) in Poland indicates that the most frequent causes of the evacuation of patients were fires and false reports about the planting of explosives (Table 1). The latter, in all cases, turned out to be thoughtless, malicious acts, whereas the fires were caused by, smoking or deliberate arson by patients in order to attract attention. Nevertheless, while the reasons for evacuation may be quite varied, evacuation plans must be prepared for many different offending circumstances and adapted to the scale of the facility, the variety of hospital wards and the health of patients.

## 4. Hospital Safety–Legal Liability

In Poland, according to the provisions of the Medical Activity Act 2011, the responsibility for the management of the medical facility, including the safety of patients and staff, lies with the health facility manager whose basic responsibilities include, among others

⮚ Designation of personnel responsible for the evacuation of staff (and patients) and combating fires.
⮚ Providing the necessary resources for emergency first-aid, firefighting, and evacuation of workers. The number of employees required to perform firefighting and evacuation activities is not specified in the regulations. The manager decides on their number and the training and equipment they should have, taking into account the type and level of risk involved. The manager is also obliged to inform other employees about who has been appointed to perform these tasks [3]. The information should include:
  – The name and surname of the designated employee(s);
  – The place where the designated employee performs their work, e.g., the department;
  – The employee’s company telephone number or other means of electronic communication.
    Employees required to perform firefighting and evacuation activities must have completed occupational health and safety training under the provisions of *the Regulation of the Minister of Economy on Training in Occupational Health and Safety* [4].
⮚ ensuring communication with external services specialized in particular in emergency first-aid, rescue and fire protection [5].

Given the above-mentioned requirements, the director of the hospital should prepare an evacuation plan for the facility, which all staff must be familiar with. Moreover, according to the Regulation on Fire Protection of Buildings, other Buildings, and Terrain, the rules of fire prevention as well as the rules of proceeding in case of fire or other local dangers should be specified in the Fire Safety Instruction Manual [6]. The Fire Safety Manual must be read and followed by all employees, regardless of their official position and type of work. The Fire Safety Manual specifies the requirements of fire protection in organizational, technical, and orderly terms, which must be taken into account subject to working in any particular building. The person preparing the Fire Safety Manual should have the appropriate qualifications for these duties. Besides, the Fire Safety Manual should be periodically updated at least once every two years, as well as after any changes in the use of the building which impact upon the change of fire protection conditions [7,8].

This obligation also requires the owner or manager of a facility intended for more than 50 permanent users to carry out a practical check of the organization and conditions of the evacuation of the entire facility at least once every two years. However, in the case of facilities in which a group of more than 50 users regularly changes concurrently, a practical check of the organization and conditions of evacuation must be carried out at least once a year. In the case of facilities containing a fire zone classified in the HH II hazard category, the scope, and area of the building covered by the practical inspection of organization and evacuation conditions must be agreed upon by the local commander of the State Fire Service [9].

Evacuation drills also allow checking, in practice, whether or not the plan has been designed correctly, including the operation of the communication and alarm system, and the cooperation of both the staff and other services involved in the evacuation process directly on site.

## 5. Hospital Evacuation Process

Each hospital should have its evacuation plan, as every medical facility is different, with varying architectural features, number of floors in the building, number of patients and their physical and mental condition, equipment and many other features. Also, each event is different. Evacuation can take place in connection with a warning that explosive materials have been planted – in this case, there are generally no obstacles in the form of smoke, fire, toxic products from combustion, problems with visibility or time pressure. Considering the various factors mentioned above, it is not possible to develop a predetermined uniform procedure for all facilities. However, each evacuation plan should have the same main purpose – to evacuate as many people as possible from the place of danger in the shortest possible time.

An evacuation plan is created specifically for each facility, but an analysis of the scientific literature has made it possible to highlight the essential elements which should be kept in mind. The order of evacuation proceedings should be similar in all facilities and follow the below-listed procedures: [10,11].

⮚ In the event of an emergency or when hospital personnel receives information about potential danger and the need to evacuate, the personnel shall trigger a warning alarm (sirens, lights, voice announcements by the personnel of the facility, other methods, according to the capabilities/equipment of the facility).
⮚ The fire brigade, the police, the director of the hospital, the head of the affected ward, as well as the other ward managers and the porters (and potentially other persons included in the evacuation plan), should be immediately notified of the event.
⮚ Staff should begin the process of evacuation of patients as soon as possible, following the plan adopted by the medical facility.
⮚ When the first unit of the State Fire Service arrives at the scene of the event, they take command of the evacuation procedure. Facility personnel should cooperate with the fire service at all times and carry out the orders of the fire officer in command. It should be remembered that the State Fire Service units arriving at the site do not know the facility, therefore, the logistical support of hospital staff is essential [12].
⮚ It is assumed that about 50% of patients on the hospital premises can move around without the help of third parties. They should, therefore, be encouraged to self-evacuate. (Patients awaiting scheduled services should be discharged from the hospital and their scheduled treatment should be postponed to another date. Also, people who are in good health may be discharged home if they need to be evacuated) [13].
⮚ The remaining patients, depending on their condition, should be transported to the hospital(s) and other places indicated in the evacuation plan. The most severely ill patients should be transported in ambulances under the care of medical personnel to the receiving hospital(s) with whom the respective medical facility has a cooperation agreement in case of evacuation. Patients who do not require medical transport may be transported to a safe place such as a hotel or school boarding house through city buses, taxis or other means of transport with which the evacuated medical entity has an agreement for emergency support.
⮚ If, after the evacuation of all patients, it is possible for the medical personnel or technical/administrative staff to return safely, they should consider the feasibility of evacuating high-value medical equipment. Medical equipment can be extremely expensive and often fragile, it is important to take action to recover and protect it where feasible. Of course, this is only possible if it does not endanger the life and health of the staff. The most expensive equipment should be recovered first. Priority should be given to items that are lightweight and easy to carry [14].

Until the emergency services arrive, it is the director of the facility who is responsible for the safety of all users of that facility. It is their responsibility to decide whether to leave the emergency area and go to a safer place. However, upon arrival at the scene of the State Fire Service, the chief commander of the rescue operation is the most senior and experienced firefighter and becomes the Rescue Leader [15]. The Rescue Leader has to carry out detailed reconnaissance of the scene of the incident, to calculate the forces and means at one’s disposal, and to decide whether these forces are sufficient or whether additional assistance needs to be called for. The management (command) of rescue operations whose size or range exceeds the capabilities of provincial-level rescue forces is taken over by the Chief Commander of the State Fire Service or an officer authorized by them [16].

Where available forces and resources are insufficient, the evacuation of the health care facility can be further supported by the Armed Forces, in particular the Territorial Defence Force. The consent for assigning forces and resources of the Polish Armed Forces to support public administration in a crisis situation is given by the Minister of National Defence, at the request of the provincial governor. The army’s support usually consists of lending power generators, securing access to water, lending tents if there is such a need to set up a military field hospital, but also using human resources to evacuate, transport the injured, and then clean up the area [17].

Also, Non-Governmental Agencies (NGO) such as the Polish Red Cross and other humanitarian organizations or foundations which would like to support the hospital materially, especially after the evacuation, can join the rescue operation. Their assistance consists mainly of equipment support in the form of mattresses, blankets, dressing materials, but also the provision of food. In turn, foundations can raise money from donors to renovate/construct the building, purchase lost equipment, apparatus, and textiles [18,19].

The institution to be notified when people suffering from infectious diseases are evacuated from the hospital or when the cause of evacuation is a dangerous infectious disease/contamination of the hospital is the local Sanitary and Epidemiological Station [20,21,22].

However, if the evacuation is caused by a bomb or terrorist attack, the hospital rescue operation and the search of the building are handled by special police and terrorism security units.

## 6. Triage Management

In a world where crisis events appear to be larger and more devastating in scope, terrorist events compete for originality and impact, and where complete hospitals are being threatened or destroyed, it is also crucial to find answers on how hospitals might adapt not only to evacuation alone but also to the unique community-wide triage management challenges that follow [23]. Plans for reverse triage must be addressed which requires actual integration of primary triage management decisions into the larger community and regional support decisions without compromising outcomes or community preparedness. Specifically, Kelen and colleagues and Burkle explored a new triage management strategy, that of reverse triage, whereby inpatients at low risk for untoward events would be discharged or transferred back into the community, giving inpatients and disaster victims equal consideration for limited inpatient resources [24]. Burkle emphasizes that “reverse triage options depend on capacity and capability of the hospital and department leadership to open a truthful and transparent dialogue with community leaders to ensure that sensible triage management options decisions are made during crisis events and are fully understood” [25]. These triage management options defined under reverse triage must not represent altered standards of care because critical life-saving resources are not reallocated. Those transferred back into the community will benefit from a system-wide program to ensure that no care or safety is compromised, guaranteeing that multiple checks and balances are incorporated into the program which is transparent and open to moral, ethical, and legal review.

## 7. Strengths, Limitations and Requirements for Future Research

This is one of the first study to assess the evacuation from healthcare facilities in Poland. Our findings may help identify discussion points on disaster planning, including preparedness, response and recovery measures, applicable to future events requiring mass evacuation.

Evacuation check-up data for 2012–2019 from the healthcare facilities were evaluated. Check-ups were provided only at the retrospective analysis incorporating media, legal acts and a literature review.

Studies and calls for further research on post-evacuation mortality and morbidity have followed the large majority of major crises. For example, the evacuees from hospitals impacted by a major earthquake in Japan showed a high prevalence of deep vein thrombosis [26], hypertension [27], atrial fibrillation [28], traumatic stress [29], and loss of life during evacuation including excess mortality among elderly evacuees [30,31,32]. Studies have also cited the risk trade-off between radiation exposure and evacuation after a nuclear power plant accident confusion perpetrated because of the loss of charts, loss of personal and medical information resulting in confusion, repeated evacuations of the same patient, and pre-crisis decisions on what authority, the medical community versus the city or state should be in control of mass evacuation from hospitals and collapse of medical services [33,34,35].

## 8. Conclusions and Recommendations

Preparing an evacuation plan for a medical facility, making it known to all staff, and regularly conducting evacuation drills is a necessary prerequisite for ensuring the safety of patients and staff.

The performance of evacuation drills should not be avoided, as only practiced procedures provide an opportunity to verify the knowledge and skills of the personnel, and to analyze the speed and effectiveness of the exercises performed. Also, to make corrections if necessary, to eliminate errors and, above all, to acquire habits that promote proper behavior.

The coordinated evacuation of a hospital requires a well thought out strategy and good preparation, and to achieve this, exercises must be carried out beforehand [36,37,38]. Evacuation preparedness education in practice settings should include more hands-on disaster preparation exercises, more “low-tech” options to address power loss, and specific policies on disaster roles. Evacuation training is a simulation of a real-life threat, but these exercises are never able to fully reflect the development of an actual real-life threat or people’s behavior. Evacuation drills involving only those wards of the hospital where the least acute or uncomplicated patients are hospitalized are a mistake, as their evacuation will probably be the easiest. It is important to practice the process of evacuation of the most complex patients, e.g. in the anesthesiology and intensive care units, as well as during surgery using high fidelity mannequins capable of mimicking a variety of acutely ill patients’ representative of the typical patient population in the facility. The staff will have the possibility to practice their roles, check the time needed to evacuate patients, plan the division of roles and responsibilities, and draw conclusions about what has been done wrong or incorrectly, what has been forgotten and what can and should be improved. This is done so that in an authentic situation, chaos does not occur, but good, well thought out cooperation becomes the standard of practice [39,40]. Unfortunately, according to the data analysis, the evacuation drills of hospitals usually involve only one ward, and typically that of the most physically and mentally fit patients.

Evacuation decision making for hospitals can be improved by ensuring hospital emergency plans address hazards and include explicit thresholds that, if exceeded, would trigger an evacuation. Comparative risk assessments that inform decision making would be enhanced by improved collection, analysis, and communication of data on morbidity and mortality associated with evacuation versus sheltering-in-place of hospitals. Also, administrators and public officials can improve their preparedness to make evacuation and shelter-in-place decisions by practicing the use of decision-making tools during training and exercises [41,42]. 

However, it should be remembered that the most important element in the evacuation process is a human. All activities related to the integration of security systems are designed to guarantee human evacuation in safe conditions.

## Figures and Tables

**Table 1:** Retrospective data on the cause of evacuation for a sample of hospital facilities (HH II facilities) in Poland.

Date, Location	HH II Facility	Cause	Additional Information
17/06/2013, Wonieść	Hospital for the mentally and neurotically ill	Fire	Around 5.30 a.m., the roof caught fire.
25/06/2013, Throughout Poland	Public institutions throughout the country, including hospitals	Report that explosives had been planted	Several dozen public institutions across the country received e-mails with the message: “at 12:00 the building will be blown up.”
31/07–01/08/2013, Kwidzyń	Hospital	Report that explosives had been planted	Phone call message: “there is a bomb in the hospital. This is not a joke. I want a million zloty (Polish currency) or everything will blow up.”
20/08/2014, Kielce	Psychiatric hospital	Fire	Fire broke out mid-day causing a mattress fire, probably started by a patient.
20/10/2014, Dąbrowa Górnicza	Hospital	Fire	After midnight, a fire resulted from a patient lighting a cigarette. A 70-year-old man took off his oxygen mask to smoke resulting in the explosion of the oxygen cylinder.
10/09/2015, Oświęcim	Hospital	Fire	The perpetrator, a 50-year-old patient brought to the emergency ward in a state of alcohol intoxication. Setting the cubicle curtains on fire with a lighter.
19/09/2016, Puławy	Hospital	Fire	At 10:30 p.m., fire occurred probably set by a 50-year-old patient.
22/09/2016, Lublin	Hospital	Fire	Crane engine room fire from a short circuit of the electrical system. At the time of the event, all fire protection devices, resistance doors separating zones, had passed inspection. Thanks to the applied technical solutions and the efficiency of technical and administrative services, the danger was noticed and reported quickly, making it possible to reduce the range and losses to a minimum.
28/12/2017, Pruszków	Hospital	Report that explosives had been planted	The police report about a bomb being planted in a local hospital.
24-25/02/2018, Warszawa	Pediatric hospital	Fire	The fire occurred in the anesthesiology and intensive care unit. 60 people evacuated, no casualties.
19/06/2018, Łódź	Hospital	Report that explosives had been planted	Midday anonymous call to the nurse’s duty room claiming an explosive device was placed in the surgical ward. Patients evacuated immediately. Pyrotechnicians search of the whole building failed to find any; no explosive device.
05/07/2018, Warszawa	Hospital	Report that explosives had been planted	A male phoned the hospital twice during the time that a well-known politician was a patient, stating that a bomb had been planted. No explosive device was found.
03/08/2019, Miszewo Murowane	Social Welfare Home	Fire	10 p.m., roof fire.
31/08/2019, Lublin	Social Welfare Home	Fire	2 a.m., roof fire.

Source: Own compilation based on a retrospective analysis of data on the reasons for the evacuation of HH II facilities.
